# Development of the Medial Longitudinal Arch of the Foot in Czech Pre- and Primary School Children—A Cross-Sectional and Longitudinal Approach

**DOI:** 10.3390/children12101407

**Published:** 2025-10-17

**Authors:** Jakub Novák, Jan Novák, Anna Vážná, Petr Sedlak

**Affiliations:** 1 Department of Anthropology and Human Genetics, Faculty of Science, Charles University, Viničná 7, 128 00 Prague, Czech Republic; 2 Division of Child Health Promotion, Department of Hygiene, Third Faculty of Medicine, Charles University, Ruská 87, 100 00 Prague, Czech Republic

**Keywords:** medial longitudinal arch, foot development, children

## Abstract

**Background/Objectives**: The medial longitudinal arch (MLA) is initially masked by a fat pad that makes the foot appear flat. In preschool age, this fat pad resorbs, and the arch becomes more defined. The exact age at which the arch attains its final form remains uncertain due to high inter-individual variability and differing assessment methods, which complicates the distinction between physiological development and potential abnormalities. Moreover, commonly used classification terms such as “flat” or “normal” do not adequately reflect the developmental progression and may be misleading in young children. This study aimed to describe the MLA developmental patterns and propose an adjusted classification terminology to improve clinical differentiation between feet undergoing normal developmental changes and cases requiring intervention. **Methods**: The present study employs both cross-sectional (285 children aged 4.00–8.99 years) and longitudinal (50 children measured annually between ages 4–6) designs. Foot dimensions were assessed using standard anthropometry, and the MLA was assessed via podograms using the Chippaux–Smirak index (CSI). To better reflect the developmental nature of the MLA, the arch was categorized as “formed” and “unformed”. Cross-sectional data were analyzed with ANOVA and visualized using LOESS regression, longitudinal data with linear mixed models, and relationships between CSI and foot dimensions with Spearman’s correlation. **Results**: MLA development showed significant changes up to age 6, with the most pronounced changes occurring between ages 4 and 5 and slowing thereafter. Children with an unformed arch at age 4 exhibited a steeper developmental trajectory than those with an already advanced arch form. Correlations between arch shape and foot dimensions were statistically significant but weak. No significant between-sex differences were observed. **Conclusions**: The timing of the most pronounced phase of medial longitudinal arch (MLA) development varies between individuals and is typically completed by 6 years of age, with no sex-dependent differences. Age 6 therefore represents a practical milestone for reliable clinical assessment, since earlier classifications risk misinterpreting normal developmental variation as pathology.

## 1. Introduction

The medial longitudinal arch (MLA) of the foot begins to develop with the onset of independent standing and walking, which typically occurs around the age of one year [1,2]. At this stage, the arch is covered by a characteristic fat pad that protects the developing skeletal structures from excessive loading, giving the foot a flat appearance [1,2]. Ossification of the MLA skeletal structures begins 1 year after birth and continues until approximately 5 years of age [1,2,3]. During this period, the fat pad gradually resorbs, and the arch becomes more apparent [2,4].

Despite these established developmental stages, there is no consensus regarding the age at which the MLA attains its final form. This uncertainty reflects both substantial inter-individual variability, as children of the same age may exhibit markedly different arch shapes even at the onset of walking [1], and the diversity of assessment methods reported in the literature, many of which are not directly comparable [5,6].

A clearer understanding of the typical developmental trajectory and timing of MLA formation could help address these challenges. Defining the age range within which the arch form most commonly stabilizes would therefore enhance clinicians’ ability to distinguish physiologically developing feet from those that deviate from expected patterns, independent of the arch assessment method used.

Current approaches to MLA classification also face conceptual limitations. Feet are most commonly classified into broad categories such as “flat”, “normal”, or “high”, based on method-specific criteria and thresholds [5]. However, such categorization does not adequately reflect the ontogenetic development of the MLA. The use of the term “normal” in early childhood is particularly problematic, as most healthy, typically developing children initially exhibit “physiologically flat feet” [5]. As noted by Uden et al. [5], this terminology is more appropriate for evaluating already fully developed arches. For this reason, the designation of MLA in early childhood would benefit from revised terminology that reflects its ongoing development.

Against this background, the present study aims (1) to describe the developmental patterns and dynamics of the MLA in preschool- and primary school-aged children and (2) to propose an adjusted classification terminology that reflects that the MLA is in an active stage of development. The ultimate goal is to improve clinical differentiation between feet undergoing normal developmental changes and those that may warrant closer monitoring or intervention.

## 2. Materials and Methods

This study utilized both cross-sectional and longitudinal observations. The cross-sectional sample comprised 285 healthy children aged 4.00 to 8.99 years (153 boys, 132 girls). The longitudinal sample consisted of annual measurements collected over three consecutive years, from ages 4 to 6. To ensure comparability, only children in the same age group for all three consecutive measurements were included (*n* = 50; 27 boys, 23 girls). Children were divided into age groups according to World Health Organization recommendations [7].

Participants were recruited through cooperation with the management of participating kindergartens and primary schools. The children’s legal guardians were informed about the study aims and procedures. Participation was entirely voluntary and required the guardian’s written informed consent authorizing their child’s involvement in the study. The overall response rate was approximately 73%. Registered children were measured in kindergartens and primary schools in Prague between the years 2018 and 2025. Recruitment procedures, measurement protocols, and equipment were consistent throughout the study period. We aimed to include all eligible children to mitigate selection bias. All participants lived in urban environments within Prague, the capital of the Czech Republic, providing comparable environmental conditions. To ensure confidentiality, all data were anonymized. The study was approved by the Institutional Review Board of the Faculty of Science, Charles University, Prague (IRB approval number 2017/23) and adhered to the Declaration of Helsinki (Fortaleza amendment).

Anthropometric measurements were conducted by trained personnel using the standardized techniques of Martin and Saller [8,9,10]. Body height was measured with a portable stadiometer (Trystom, s.r.o., Olomouc, Czech Republic) to an accuracy of 1 mm, while body weight was obtained using a personal step scale (Hyundai Corporation, Seoul, Republic of Korea) with a precision of 0.1 kg. Body mass index (BMI) was calculated as the ratio of weight (kilograms) to body height squared (meters). BMI z-score was calculated in RůstCZ software (version 2.3) [11], which utilizes Czech Republic reference data from the 6th Nationwide Anthropological Survey of Children and Adolescents [12]. Children with a BMI z-score greater than +1 were classified as with excess weight.

To complement the MLA development, basic foot dimensions were monitored. Foot length was measured as the distance from *pternion* (the most posterior point of the heel) to *akropodion* (the most anterior point of the first toe) [8]. Foot width was measured as the distance between *metatarsale tibiale* (the most prominent medial point of the first metatarsal head) and *metatarsale fibulare* (the most prominent lateral point of the fifth metatarsal head) [8]. Both dimensions were measured, using a cephalometer, to the nearest 2 mm. Relative foot length was calculated as a percentage of body height using this formula: (foot length/body height) × 100. Foot width was normalized to foot length by calculating the foot index: (foot width/foot length) × 100 [1]. Measurements were conducted only on the right foot, which was randomly selected prior to the start of the study.

**Figure 1:**
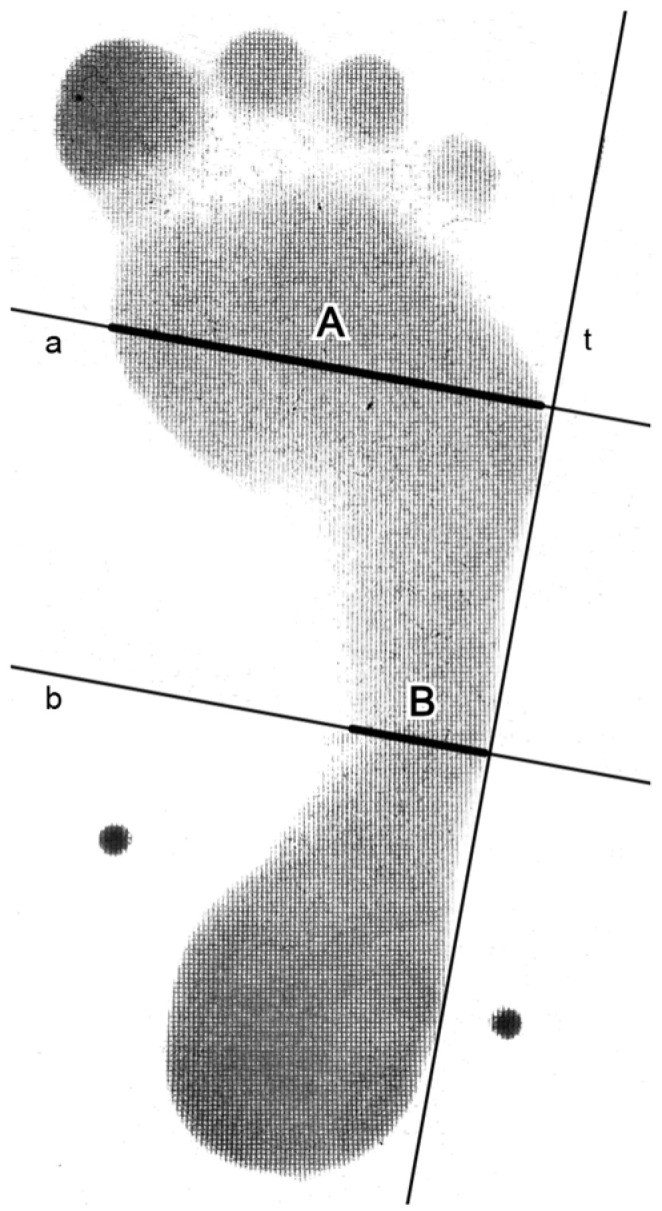
Chippaux–Smirak index. (**A**)—widest forefoot width; (**B**)—narrowest midfoot width; CSI = (B/A) × 100 (adapted from Šmiřák [14]). In the figure, *a* represents the perpendicular line to the widest forefoot width, *b* represents the perpendicular line to the narrowest midfoot width, and *t* indicates the tangent line.

The MLA was assessed through podogram analysis. Podograms were obtained in 100% weight-bearing position using a Bauerfeind Pedoprint podograph (Bauerfeind AG, Zeulenroda-Triebes, Germany). The resulting footprints were scanned with an Epson Perfection V19 scanner and analyzed digitally using the GeoGebra software (version 6.0.879.0) [13]. Podogram evaluation was performed using the Chippaux–Smirak Index (CSI) method [14]. The CSI was expressed as the percentage ratio of the narrowest midfoot width to the widest forefoot width, measured perpendicular to the lateral tangent of the podogram (Figure 1) [14].

All podograms were assessed by a single examiner—with a coefficient of reliability of 0.996, determined by performing between-day repeated testing—in duplicate on a pilot sample of 30 footprints [15]. Although inter-rater reliability was not directly assessed in this study, previous work by Queen et al. has reported good inter-rater reliability of the CSI [15]. The CSI method was selected due to its widespread use in international research [16,17,18,19,20,21] and its recommendation over other methods for monitoring arch development in children [16,19].

To account for the ontogenetic development of the MLA, foot classification terminology was adjusted based on the degree of arch formation. The arch was categorized as either “formed” (CSI ≤ 45%), corresponding to a “normal” foot type, or “unformed” (CSI > 45%), indicating a “flat” foot type, in accordance with the standard CSI cut-off value [14,22]. Podograms that showed no connection between the forefoot and hindfoot sections of the imprint were classified as high arched.

Statistical analyses were conducted using the R programming language (version 4.3.2) [23] and jamovi statistical software (version 2.3.28) [24]. The significance level was set at *p* < 0.05, and 95% confidence intervals (CI) were used for all statistical analyses. Homogeneity of variances was assessed using Levene’s test, and normality was tested with the Shapiro–Wilk test. Outliers were excluded based on residual z-scores exceeding ±3 standard deviations (SD). Participants with high arches (*n* = 7; 4 boys, 3 girls) were excluded from the CSI-based analyses, as the classification method applied to these cases did not provide CSI values.

Intra-rater reliability was evaluated using an intraclass correlation coefficient (ICC) based on a two-way mixed effects model with a consistency definition and single measurements [15,25]. The resulting ICC was 0.996 (CI [0.992, 0.998]).

Spearman’s rank-order correlation analysis was used to assess associations between the CSI and foot dimensions. Correlation strength was interpreted as weak (ρ ≥ 0.3), moderate (ρ ≥ 0.5), or strong (ρ ≥ 0.7) based on Spearman’s ρ absolute value [26].

One-way analysis of variance (ANOVA) was used in an exploratory manner to examine age- and sex-related differences in body height, foot dimensions, and CSI, with the goal of identifying developmental periods with notable changes. When ANOVA assumptions were met, Bonferroni correction was applied to control for multiple comparisons; if assumptions were violated, Welch’s ANOVA with Games-Howell post hoc correction was used.

Locally estimated scatterplot smoothing (LOESS) regression was used to visualize nonlinear trends in the development of CSI across age. The LOESS curves were used descriptively to complement the inferential analyses (ANOVA) by highlighting age-related trajectories and potential sex differences.

Linear mixed models were used in an exploratory manner to analyze repeated measurements of body height, foot dimensions, and CSI within individuals over time. The aim of these analyses was to identify dynamics of developmental change and potential sex or other factor differences. The statistical significance of factor effects on the dependent variables was evaluated using a fixed effect omnibus test with Bonferroni correction.

Statistical power was estimated using G*Power software (version 3.1.9.7) [27]. Post hoc power analyses were conducted based on the acquired sample size and the specifications of each statistical test. The total sample size for both the transversal and longitudinal datasets provided power > 0.8. For subgroup analyses, power ranged between 0.6 and 0.8.

## 3. Results

### 3.1. Cross-Sectional Observations

**Figure 2:**
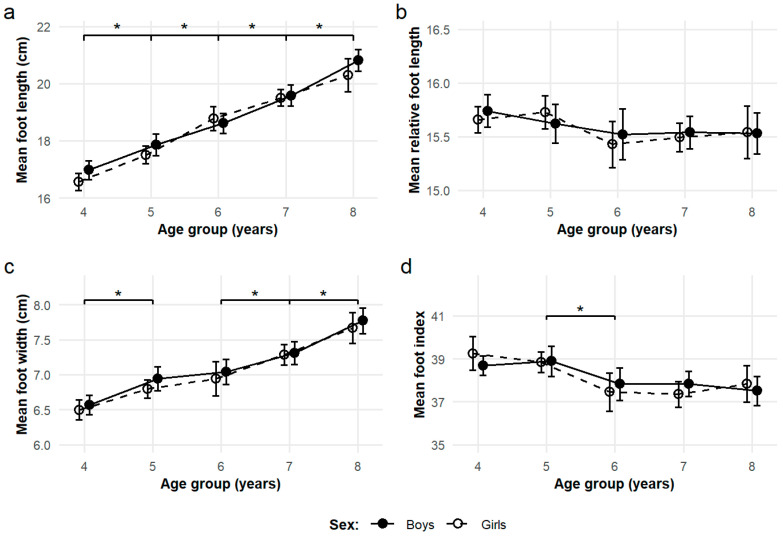
Age-related changes in foot dimensions and proportions. (**a**) Mean foot length; (**b**) mean relative foot length (foot length normalized by height); (**c**) mean foot width; and (**d**) mean foot index (foot width/length × 100) are plotted for each age group. Brackets with significance markers (*) indicate statistically significant differences between adjacent age groups for the total sample at *p* < 0.05. Error bars represent 95% CI. Sex-specific trajectories are shown to illustrate developmental trends in boys and girls. Boys are represented by filled circles and solid lines; girls by open circles and dashed lines.

Basic characteristics of the cross-sectional observations sample are presented in Table 1. Based on the BMI z-score, 9% (*n* = 26; 11 boys, 15 girls) of the children had excess weight (z-score BMI ≥ +1 SD). Sex and age group differences in body height and foot dimensions were analyzed on the total sample using ANOVA. Significant between-sex differences were observed in body height (F = 4.40, *p* = 0.037) and foot length (F = 3.94, *p* = 0.048), with boys being taller and having longer feet than girls. Across age groups, the foot length demonstrated a linear increase with age and body height. Foot width exhibited a similar trend, except between the ages of 5 and 6, where no significant difference was observed. This was reflected in a significant difference in foot index values between the 5- and 6-year-old groups (F = 10.05, *p* < 0.001). No significant differences were observed in the foot length to body height index. The developmental trends of foot dimensions and proportions are presented in Figure 2.

A correlation analysis was conducted to assess the relationship between arch shape, expressed by CSI, and foot dimensions. The results revealed statistically significant but weak correlations between CSI and foot length (ρ = −0.19, *p* = 0.001), foot index (ρ = 0.24, *p* < 0.001), and relative foot length (ρ = 0.19, *p* = 0.002), whereas foot width showed no significant association (ρ = −0.07, *p* = 0.274).

**Figure 3:**
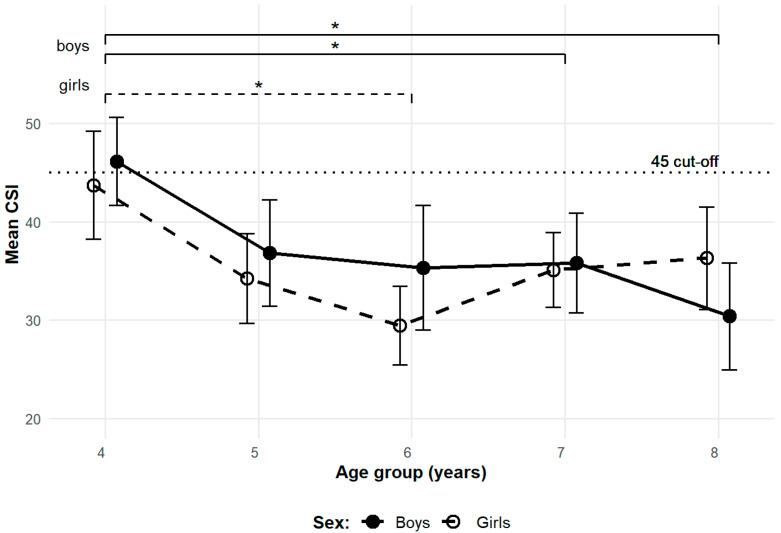
Sex specific change in mean CSI across age groups—transversal dataset. Brackets with significance markers (*) indicate significant differences at *p* < 0.05 between age groups (solid lines for boys; dashed lines for girls). Error bars represent 95% CI. The dotted line represents the CSI cut-off value distinguishing unformed and formed arches. Boys are represented by filled circles and solid lines; girls by open circles and dashed lines.

ANOVA was used to identify sex specific significant periods of MLA development based on mean CSI (Figure 3). A post hoc test with Bonferroni correction revealed significant differences in boys between ages 4–7 (t = 2.87, *p* = 0.047, CI [4.68, 15.92]) and ages 4–8 (t = 4.10, *p* < 0.001, CI [7.69, 23.77]). Despite the mean CSI of 6-year-old boys being lower than that of 7-year-old boys, no significant difference was observed between ages 4–6. The lack of a significant difference between these groups may be reflected by lower statistical power due to the limited sample size. Because of unequal variances among the age groups of girls, Welch’s one-way ANOVA with a Games-Howell post hoc test was applied, revealing a significant difference between ages 4–6 (t = 4.11, *p* = 0.001, CI [7.46, 21.04]). Although the use of different statistical tests limits direct comparison, the between sex differences were assessed on the whole sample, which met the assumptions for parametric ANOVA. A post hoc test with Bonferroni correction revealed no significant sex-based differences within the individual age groups.

**Figure 4:**
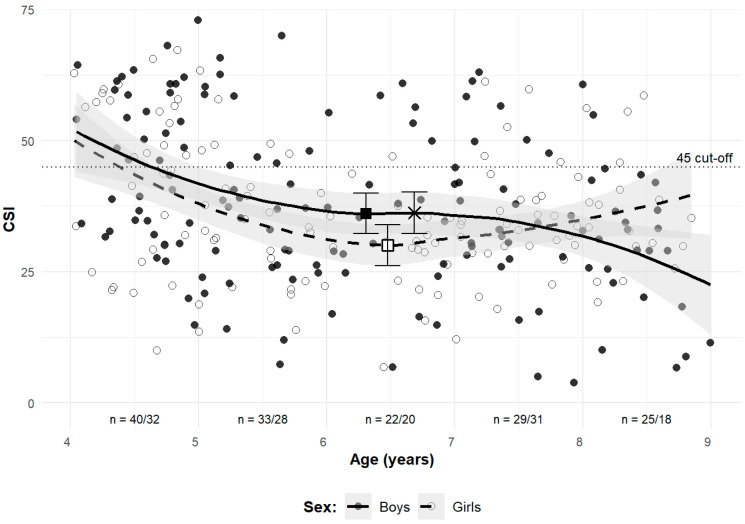
LOESS plot of CSI by age and sex. Shaded areas represent the 95% confidence interval bands for the estimated regression curves. The dotted horizontal line indicates the CSI cut-off value distinguishing unformed and formed arches. Boys are represented by filled circles and solid lines, while girls are represented by open circles and dashed lines. The filled black square indicates the first local minimum for boys, the X cross indicates the second local maximum for boys, and the empty square indicates the local minimum for girls. The “n=” labels below the *x*-axis show the number of observations for boys/girls in each 1-year age bin.

A locally estimated scatterplot smoothing (LOESS) plot was used to illustrate the continuous development of CSI with age (Figure 4). The regression curves for both sexes exhibited a similar decline in CSI until approximately age 6, followed by a plateau until age 7. After age 7, the regression curve for boys continued to decline, whereas the regression curve for girls showed an increase. The exact ages of the estimated local minima, along with their CSI values and confidence intervals, are as follows: the first local minimum for boys occurred at age 6.31 (CSI = 36.1, CI [32.2, 39.9]), and the local minimum for girls occurred at age 6.48 (CSI = 30.0, CI [26.1, 34.0]). A second local maximum for boys was observed at age 6.69 (CSI = 36.2, CI [32.3, 40.1]), after which CSI values showed a secondary decline. It should be noted that the endpoints of the regression curves may be less reliable due to potential edge effects.

### 3.2. Longitudinal Observations

Children in the longitudinal observations sample were selected to ensure that each measurement corresponded to children within the same age group. Basic characteristics of the longitudinal observations sample are presented in Table 2. Based on the BMI z-score, 10% (*n* = 5) of children had excess weight (z-score BMI > +1 SD) during the first measurement, and only 2 of them retained it through all three measurements (reported for sample characteristics purposes). A linear mixed model with a fixed effects omnibus test was applied to the total sample to analyze differences in body height and foot dimensions between sexes and across age groups. No significant between-sex differences were observed. Foot length and foot width showed a linear increase with age and body height. Significant differences were found in the foot index (F = 16.16, *p* < 0.001) and the relative foot length (F = 20.94, *p* < 0.001), both of which declined with age in a similar pattern to that observed across age groups 4–6 in Figure 2 of the cross-sectional sample.

**Figure 5:**
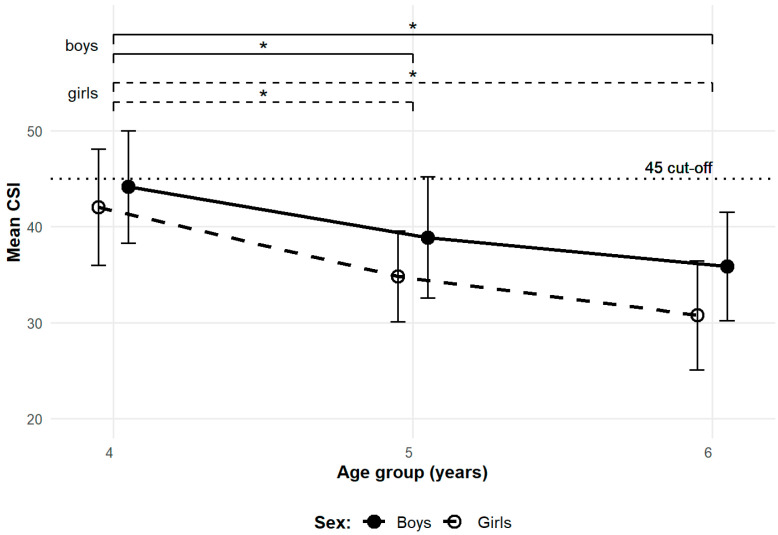
Sex specific change in mean CSI across age groups—longitudinal dataset. Brackets with significance markers (*) indicate significant differences at *p* < 0.05 between age groups (solid lines for boys; dashed lines for girls). Error bars represent 95% CI. The dotted line represents the CSI cut-off value distinguishing unformed and formed arches. Boys are represented by filled circles and solid lines; girls by open circles and dashed lines.

To determine key periods of MLA development, we compared mean CSI values across consecutive age groups for both sexes (Figure 5). The fixed effect omnibus test with Bonferroni correction revealed a significant difference between ages 4–5 and 4–6, in boys (t = 2.85, *p* = 0.019, CI [1.64, 8.90]; t = 4.48, *p* < 0.001, CI [4.66, 11.91]) and girls (t = 3.17, *p* = 0.008, CI [2.75, 11.65]; t = 4.97, *p* < 0.001, CI [6.82, 15.71]), respectively. No significant differences were found between ages 5–6 for either sex. No significant between-sex differences were observed.

We further investigated whether the dynamics of MLA development varied between children with already formed arches and those whose arches were still unformed. Based on CSI values from the first year of measurement, children were categorized into two groups using the standard CSI cut-off: the “unformed arch group” (CSI > 45) and the “formed arch group” (CSI ≤ 45). Table 3 presents the age and CSI characteristics for each group across measurements. Although children with formed arches were, on average, slightly older than those with unformed arches, the fixed-effects omnibus test revealed no statistically significant age difference between the groups. The notably high CSI standard deviations indicate substantial inter-individual variability in the progression of MLA development.

**Figure 6:**
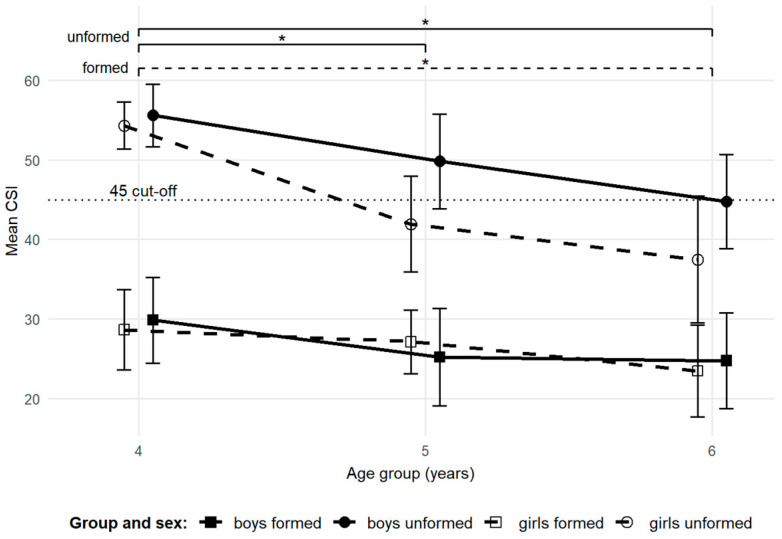
Change in mean CSI across age groups for the formed and unformed arch group. Arch form groups were classified based on their initial measurement CSI values. Brackets with significance markers (*) indicate significant differences at *p* < 0.05 between age groups (solid lines for unformed arch group; dashed line for formed arch group). Error bars represent 95% CI. The dotted line represents the CSI cut-off value distinguishing unformed and formed arches. Sex-specific trajectories are shown to illustrate developmental trends in boys and girls. Boys are represented by filled circles and solid lines; girls by open circles and dashed lines.

The fixed-effect omnibus test revealed that the CSI values differed significantly between the formed and unformed group (t = −8.74, *p* < 0.001, CI [−25.47, −16.15]). Within the formed arch group, a significant difference was observed between ages 4–5 (t = 2.87, *p* = 0.019, CI [1.63, 8.67]), with no significant between-sex difference. In the unformed arch group, CSI values differed significantly between ages 4–5 (t = 4.40, *p* < 0.001, CI [5.04, 13.13]) and between ages 4–6 (t = 6.70, *p* < 0.001, CI [9.78, 17.87]), again with no between-sex differences. Although no statistically significant difference between sexes was observed, the mean CSI for boys in the unformed arch group only slightly exceeded the cut-off for a formed arch at age 6, whereas for girls, the mean CSI had already crossed the cut-off at age 5 (Figure 6).

## 4. Discussion

This study aimed to describe the developmental patterns and dynamics of the medial longitudinal arch (MLA) of the foot during the key developmental period of pre- and primary school age, while also examining its relationship to basic foot dimensions and potential between-sex differences.

A between-sex comparison of body height and foot dimensions showed that boys were generally taller and had longer feet than girls. After adjusting foot length for body height and foot width, no significant differences between sexes were observed in the resulting proportions. This finding suggests that the apparent sex differences primarily reflect overall differences in body size, rather than differences in relative foot proportions. Comparable findings have been reported in previous studies [4], where similar adjustments of the foot dimensions also reduced apparent sex-based differences.

Across age groups, a linear increase in foot length and width was observed, except between ages 5 and 6, where no significant change in foot width was found. This was reflected by a decline in foot index values during the same period, which then remained stable at lower levels in subsequent age groups. This seeming slowdown in foot width growth relative to foot length may be an artifact of age group classification, as a longitudinal analysis of foot width did not replicate this trend. Longitudinal analysis revealed a significant decline in relative foot length and foot index with age, which paralleled the general pattern of arch development based on CSI during the observed period. However, correlation analysis between CSI and foot dimensions and proportions indicated, at most, a weak relationship, suggesting that the parallel age-related trends do not necessarily reflect a direct association between foot shape and arch formation.

For the evaluation of MLA, we redefined the standard classification terminology to emphasize its ontogenetic development. Rather than using traditional terms such as “normal” and “flat”, the arch was classified as “formed” (CSI ≤ 45%) or “unformed” (CSI > 45%) based on the standard CSI cut-off values [14,22]. This approach acknowledges that the MLA is naturally unformed (“physiologically flat”) at the onset of walking and gradually develops with age into “normal” arch shape. We further propose that the term “flat foot” should be reserved for pathological cases—where the arch remains unformed or has secondarily collapsed after the end of the developmental period.

Our analysis of mean CSI changes across age groups indicated that the most pronounced period of MLA development concludes by age 6 in both sexes. This is further supported by the LOESS plot of CSI against age, with regression curves reaching a local minimum around age 6, followed by a brief stabilization until age 7 for both boys and girls. Beyond this point, the regression curves indicated subtle sex-dependent fluctuations in MLA shape.

No statistically significant between-sex differences in MLA development were observed in our cohort, which contrasts with general trends reported in the literature, where boys often exhibit less developed arches during the same observed age [28,29,30,31,32]. Exploratory inspection, however, revealed minor, non-significant patterns. In boys, the mean CSI showed a slight increase at age 7 before decreasing at age 8, a pattern also noted by Forriol et al. [17] in Spanish boys. In girls, CSI increased between 6 and 8 years, mirroring a trend also reported by Forriol et al. [17] in 7 and 8-year-old Spanish girls. These patterns were observed despite methodological differences across studies [16,31]. Chang et al. [31] suggested that increased obesity in 8-year-old children may play a role, whereas Onodera et al. [16] attributed observed declines to sample heterogeneity, as they had to merge 7- and 8-year-old participants into a single group due to limited sample size. Given that these trends were not statistically significant in our cohort, further investigation would be needed to determine whether these patterns represent true sex-specific developmental differences or merely reflect the variability within the sample.

Longitudinal observations were used to analyze the dynamics of MLA development. The steepest, statistically significant decline was observed between ages 4 and 5. The decline between ages 5 and 6 was no longer statistically significant, which could indicate a reduction in developmental dynamics, as also reflected in our cross-sectional analysis. This trend is consistent with Tong and Kong’s longitudinal observations in Singaporean children between ages 7 and 9 [32]. Despite using a different footprint method, the authors reported overall stability in MLA measures, with only subtle, statistically weak changes in boys and no measurable changes in girls. In contrast, Bosch et al. illustrated minor fluctuations in MLA shape in German children up to age 9 [33]. However, these between-age differences were not statistically tested, and this interpretation relies mainly on visual inspection of the plotted data. Taken together, these findings suggest that MLA development slows considerably after age 6, with any subsequent physiological changes in MLA shape expected to be subtle, thereby narrowing the developmental window, which some studies had previously reported to extend across the first decade of life [6,16,34].

Further analysis was conducted to determine whether the arch formation dynamics differed between children with unformed arches and those with already formed arches at the time of first measurement. Although children with formed arches were, on average, slightly older than those with unformed arches, the age difference was not significant. Children who entered the study with unformed arches experienced a significant decrease in mean CSI between ages 4 and 5, reflecting pronounced arch formation during this period. By contrast, children with already formed arches showed only a slight decrease, significant across the broader period from 4 to 6 years. The absence of significant change in either group from 5 to 6 years supports the previously noted deceleration in MLA formation dynamics. These findings suggest that timing of the most pronounced developmental period varies among individuals but generally occurs before the age of 6.

Although no significant between-sex differences were found between the two arch form groups, the mean CSI of girls in the unformed arch group crossed the formed arch cutoff at 5 years—about one year earlier than boys, whose mean CSI only approached the cutoff by age 6. This pattern may indicate a tendency toward earlier MLA formation in girls, but given the limited subgroup sizes, such differences should be considered exploratory rather than conclusive. Previous studies have reported similar trends: Unger and Rosenbaum observed more formed arches in girls during early ambulation [35], and Mickle et al. noted that preschool-aged girls had a thinner midfoot fat pad than boys [4]. Whether these observations reflect true sex-specific developmental differences or simply variability within small samples remains uncertain.

Our results are based on footprint analyses, which provide only a static, two-dimensional assessment of the longitudinal arch. Still, the developmental patterns observed in our study appear broadly consistent with those reported in recent international standards for foot posture assessment [36]. In the referenced study, the foot posture was evaluated using the FPI-6 method, which offers a more comprehensive, multi-planar assessment [36]. Nevertheless, it should be emphasized that this comparison relies primarily on visual inspection of the plotted data and has not been validated against clinical outcomes or the FPI-6 method.

Another alternative for assessing the MLA is radiography, which provides more detailed, multi-planar measures and is often considered more objective [37]. Nevertheless, footprint-based methods and the FPI-6 remain favorable for routine clinical use due to their simplicity, safety, and suitability for repeated screening and longitudinal monitoring of arch development in children. Some studies even report comparable performance of footprint analysis and radiography in evaluating the MLA [38,39].

In addition, shoe-wearing habits should be considered in the context of longitudinal arch development, as they represent an important external factor influencing both the function and morphology of the foot [40,41]. Footwear can modify the biomechanics and kinematics of the foot in various ways [42,43,44], potentially affecting its natural development. Across different ethnicities, children who regularly wear shoes exhibit a higher prevalence of unformed or flattened arches compared with those who remain habitually barefoot [43,45,46,47,48]. For instance, Aibast et al. [49] reported that among the Kalenjin tribe in Kenya, children and adolescents who combined high levels of physical activity with barefoot walking developed more pronounced longitudinal arches and stronger foot ligaments, tendons, and muscles than those who routinely wore shoes. In European contexts, where shoe use is common, it is likely that the children in the present study developed their arches in a predominantly shod environment, although this could not be directly verified.

Several limitations of this study should be acknowledged. Lower statistical power in subgroup analyses may have reduced the ability to detect subtle effects or small differences. The extended data collection period may have affected the homogeneity of the cohort in the cross-sectional sample. Measurements were conducted only on the right foot, which prevents assessment of potential asymmetries between limbs. The Chippaux–Smirak Index provides only a two-dimensional, static proxy for the medial longitudinal arch. Finally, body mass index (BMI) was not adjusted for in the analyses.

## 5. Conclusions

This study investigated the timing and pattern of medial longitudinal arch development in pre- and primary-school children, including potential between-sex differences. Our results indicate that the most pronounced phase of medial longitudinal arch formation is largely complete by approximately 6 years of age in both sexes, after which the rate of change declines sharply, with only subtle, clinically minor alterations expected up to 9 years. We further observed considerable inter-individual variability in the arch formation dynamics between 4 and 6 years of age.

These findings carry important clinical implications. Because arch morphology remains actively developing prior to around 6 years of age, definitive clinical classification of a child’s foot as “flat” or “normal” should be avoided during this period, regardless of the assessment method used. Instead, pediatric medial longitudinal arch status should be interpreted within the context of ongoing ontogenetic development, with feet in early childhood assessed as either “formed” or “unformed” until the arch has completed its principal phase of formation. This approach more accurately reflects physiological development and reduces the risk of misclassifying a healthy, developing foot as pathological and identifies six years of age as the point from which closer monitoring or intervention may be warranted.

## Figures and Tables

**Table 1:** Number of participants and sample characteristics for each age group and sex—transversal dataset ^a^.

Variable	Sex	Age Group				
		4.00–4.99	5.00–5.99	6.00–6.99	7.00–7.99	8.00–8.99
***n***	boys	40	33	23	32	25
	girls	32	30	20	31	19
Age (years)	boys	4.6 (0.3)	5.4 (0.3)	6.5 (0.3)	7.4 (0.3)	8.4 (0.3)
	girls	4.5 (0.3)	5.4 (0.3)	6.6 (0.3)	7.5 (0.3)	8.4 (0.3)
Body height (cm)	boys	107.6 (4.8)	114.4 (6.1)	119.9 (3.7)	126.0 (5.6)	134.0 (5.1)
	girls	105.8 (4.9)	111.7 (3.8)	121.7 (5.2)	125.9 (3.9)	130.5 (6.3)
BMI (kg/m^2^)	boys	15.5 (1.5)	15.5 (1.2)	14.9 (0.9)	15.8 (1.5)	16.0 (1.3)
	girls	15.4 (1.2)	15.3 (1.1)	14.9 (1.3)	16.0 (1.8)	16.5 (1.8)
BMI z-score ^b^	boys	−0.1 (0.9)	0.0 (0.8)	−0.4 (0.6)	−0.0 (0.9)	−0.0 (0.7)
	girls	0.0 (0.8)	0.0 (0.7)	−0.4 (0.8)	0.1 (0.8)	0.1 (0.9)
CSI	boys	46.1 (14.4)	36.8 (15.8)	35.3 (15.4)	35.8 (14.6)	30.4 (13.9)
	girls	43.7 (15.9)	34.2 (12.7)	29.5 (9.1)	35.1 (10.8)	36.3 (11.6)
Foot length (cm)	boys	17.0 (1.1)	17.9 (1.1)	18.6 (0.9)	19.6 (1.1)	20.8 (1.0)
	girls	16.6 (0.9)	17.5 (0.9)	18.8 (0.9)	19.5 (0.8)	20.3 (1.3)
Foot width (cm)	boys	6.6 (0.4)	6.9 (0.5)	7.0 (0.4)	7.3 (0.5)	7.8 (0.5)
	girls	6.5 (0.4)	6.8 (0.4)	6.9 (0.6)	7.3 (0.4)	7.7 (0.5)
Foot index	boys	38.7 (1.4)	38.9 (2.1)	37.8 (1.8)	37.8 (1.7)	37.5 (1.7)
	girls	39.3 (2.3)	38.8 (1.3)	37.5 (2.0)	37.3 (1.7)	37.8 (1.9)
Relative foot length	boys	15.7 (0.5)	15.6 (0.5)	15.5 (0.6)	15.5 (0.4)	15.5 (0.5)
	girls	15.7 (0.4)	15.7 (0.4)	15.4 (0.5)	15.5 (0.4)	15.5 (0.5)

^a^ Data are presented as mean values with standard deviations (SD) in brackets. ^b^ BMI z-score was calculated using the freely available software RůstCZ [11], which uses data from the Czech Republic reference [12]. BMI = body mass index. CSI = Chippaux–Smirak index (unformed arch = CSI > 45, formed arch = CSI ≤ 45). Foot index = (foot width/foot length) × 100. Relative foot length = (foot length/body height) × 100.

**Table 2:** Number of participants and sample characteristics for each age group and sex—longitudinal dataset ^a^.

Variable	Sex	Measurement ^b^ (Age Group)		
		1 (4.00–4.99)	2 (5.00–5.99)	3 (6.00–6.99)
***n***	boys	27	27	27
	girls	23	23	23
Age (years)	boys	4.5 (0.3)	5.5 (0.3)	6.5 (0.3)
	girls	4.5 (0.3)	5.5 (0.3)	6.5 (0.3)
Body height (cm)	boys	108.3 (6.1)	115.0 (6.4)	121.4 (6.8)
	girls	105.8 (5.1)	112.2 (5.2)	118.7 (5.8)
BMI (kg/m^2^)	boys	15.4 (1.1)	15.2 (1.2)	15.5 (1.8)
	girls	15.1 (1.1)	15.1 (1.3)	15.0 (1.4)
BMI z-score ^c^	boys	−0.1 (0.8)	−0.1 (0.7)	−0.1 (0.8)
	girls	−0.2 (0.8)	−0.1 (0.8)	−0.3 (0.8)
CSI	boys	44.2 (15.5)	38.9 (16.7)	35.9 (15.0)
	girls	42.0 (14.8)	34.8 (11.6)	30.8 (13.9)
Foot length (cm)	boys	17.2 (1.1)	18.0 (1.2)	18.7 (1.0)
	girls	16.6 (1.0)	17.5 (1.1)	18.4 (1.1)
Foot width (cm)	boys	6.6 (0.4)	6.9 (0.5)	7.1 (0.5)
	girls	6.5 (0.4)	6.8 (0.4)	7.1 (0.4)
Foot index	boys	38.5 (1.4)	38.6 (1.7)	37.8 (1.5)
	girls	39.3 (1.4)	38.9 (1.4)	38.4 (2.0)
Relative foot length	boys	15.8 (0.4)	15.6 (0.5)	15.6 (0.5)
	girls	15.7 (0.5)	15.6 (0.4)	15.5 (0.4)

^a^ Data are presented as mean values with standard deviations (SD) in brackets. ^b^ Each measurement corresponds to children in the same age group (in brackets). ^c^ BMI z-score was calculated using the freely available software RůstCZ [11], which uses data from the Czech Republic reference [12]. BMI = body mass index. CSI = Chippaux–Smirak index (unformed arch = CSI > 45, formed arch = CSI ≤ 45). Foot index = (foot width/foot length) × 100. Relative foot length = (foot length/body height) × 100.

**Table 3:** Mean age and CSI of groups classified based on their initial measurement CSI values ^a^.

	Variable	Sex	Measurement		
			1	2	3
Unformed arch group	*n*	boys	15	15	15
		girls	12	12	12
	Age (years)	boys	4.4 (0.2)	5.4 (0.2)	6.4 (0.2)
		girls	4.4 (0.3)	5.4 (0.3)	6.4 (0.3)
	CSI	boys	55.6 (7.7)	49.8 (11.7)	44.8 (11.7)
		girls	54.3 (5.2)	41.9 (10.6)	37.5 (14.1)
Formed arch group	*n*	boys	12	12	12
		girls	11	11	11
	Age (years)	boys	4.7 (0.3)	5.7 (0.3)	6.7 (0.3)
		girls	4.6 (0.3)	5.6 (0.3)	6.6 (0.3)
	CSI	boys	29.9 (9.5)	25.2 (10.8)	24.8 (10.7)
		girls	28.6 (8.5)	27.1 (6.8)	23.5 (9.7)

^a^ Data are presented as mean values and standard deviations (SD) in brackets. CSI = Chippaux–Smirak index (unformed arch = CSI > 45, formed arch = CSI ≤ 45).

## Data Availability

The original data presented in the study are openly available in Zenodo at https://doi.org/10.5281/zenodo.16813083 (accessed on 12 August 2025).

## Abbreviations

The following abbreviations are used in this manuscript:

MLA	Medial Longitudinal Arch
BMI	Body Mass Index
CSI	Chippaux–Smirak Index
CI	Confidence Intervals
SD	Standard Deviation
ICC	Intraclass Correlation Coefficient
ANOVA	Analysis of Variance
LOESS	Locally Estimated Scatterplot Smoothing
